# Single Nucleotide Polymorphism Discovery in Bovine Pituitary Gland Using RNA-Seq Technology

**DOI:** 10.1371/journal.pone.0161370

**Published:** 2016-09-08

**Authors:** Chandra Shekhar Pareek, Rafał Smoczyński, Haja N. Kadarmideen, Piotr Dziuba, Paweł Błaszczyk, Marcin Sikora, Paulina Walendzik, Tomasz Grzybowski, Mariusz Pierzchała, Jarosław Horbańczuk, Agnieszka Szostak, Magdalena Ogluszka, Lech Zwierzchowski, Urszula Czarnik, Leyland Fraser, Przemysław Sobiech, Krzysztof Wąsowicz, Brian Gelfand, Yaping Feng, Dibyendu Kumar

**Affiliations:** 1 Division of Functional Genomics in Biological and Biomedical Research, Centre for Modern Interdisciplinary Technologies, Nicolaus Copernicus University, Torun, Poland; 2 Department of Large Animal Sciences, Faculty of Health and Medical Sciences, University of Copenhagen, Copenhagen, Denmark; 3 Ludwik Rydygier Collegium Medicum, Institute of Forensic Medicine, Department of Molecular and Forensic Genetics, The Nicolaus Copernicus University, Bydgoszcz, Poland; 4 Institute of Genetics and Animal Breeding of the Polish Academy of Sciences, Jastrzebiec, Poland; 5 Faculty of Animal Bio-engineering, University of Warmia and Mazury in Olsztyn, Olsztyn, Poland; 6 Faculty of Veterinary Medicine, University of Warmia and Mazury in Olsztyn, Olsztyn, Poland; 7 Waksman Institute of Microbiology, Rutgers, The State University of New Jersey, Piscataway, New Jersey, United States of America; University of Florida, UNITED STATES

## Abstract

Examination of bovine pituitary gland transcriptome by strand-specific RNA-seq allows detection of putative single nucleotide polymorphisms (SNPs) within potential candidate genes (CGs) or QTLs regions as well as to understand the genomics variations that contribute to economic trait. Here we report a breed-specific model to successfully perform the detection of SNPs in the pituitary gland of young growing bulls representing Polish Holstein-Friesian (HF), Polish Red, and Hereford breeds at three developmental ages viz., six months, nine months, and twelve months. A total of 18 bovine pituitary gland polyA transcriptome libraries were prepared and sequenced using the Illumina NextSeq 500 platform. Sequenced FastQ databases of all 18 young bulls were submitted to NCBI-SRA database with NCBI-SRA accession numbers SRS1296732. For the investigated young bulls, a total of 113,882,3098 raw paired-end reads with a length of 156 bases were obtained, resulting in an approximately 63 million paired-end reads per library. Breed-wise, a total of 515.38, 215.39, and 408.04 million paired-end reads were obtained for Polish HF, Polish Red, and Hereford breeds, respectively. Burrows-Wheeler Aligner (BWA) read alignments showed 93.04%, 94.39%, and 83.46% of the mapped sequencing reads were properly paired to the Polish HF, Polish Red, and Hereford breeds, respectively. Constructed breed-specific SNP-db of three cattle breeds yielded at 13,775,885 SNPs. On an average 765,326 breed-specific SNPs per young bull were identified. Using two stringent filtering parameters, i.e., a minimum 10 SNP reads per base with an accuracy ≥ 90% and a minimum 10 SNP reads per base with an accuracy = 100%, SNP-db records were trimmed to construct a highly reliable SNP-db. This resulted in a reduction of 95,7% and 96,4% cut-off mark of constructed raw SNP-db. Finally, SNP discoveries using RNA-Seq data were validated by KASP^™^ SNP genotyping assay. The comprehensive QTLs/CGs analysis of 76 QTLs/CGs with RNA-seq data identified *KCNIP4*, *CCSER1*, *DPP6*, *MAP3K5* and *GHR* CGs with highest SNPs hit loci in all three breeds and developmental ages. However, *CAST* CG with more than 100 SNPs hits were observed only in Polish HF and Hereford breeds.These findings are important for identification and construction of novel tissue specific SNP-db and breed specific SNP-db dataset by screening of putative SNPs according to QTL db and candidate genes for bovine growth and reproduction traits, one can develop genomic selection strategies for growth and reproductive traits.

## Introduction

In recent years, the development of RNA-seq technology has completely revolutionized the way of thinking in molecular biology, and allowed a more detailed insight into gene expression by examining expression level, sequence variation, gene structure, and strand-specificity simultaneously [1–4]. The bovine genome is about 3 GB (3 billion base pairs), and contains approximately 22,000 genes, of which 14,000 are common to all mammalian species. The genome assembly of a Hereford cow produced by the Baylor College of Medicine Human Genome Sequencing Centre (BCM-HGSC) has now allowed years of work in cattle QTL mapping to be associated with genes and other genomic features [5–6]. This high throughput genome sequencing and identification of abundant amount of DNA variants for the bovine genome have significantly facilitated the analysis of genetic variation among cattle breeds. However, very little is known regarding the transcriptomic expression of genes in cattle and the variation between breeds. However, very little is known regarding the SNP-driven transcriptomic expression of genes in cattle and the tissue level database of SNPs (SNP-db) that associate with expressed genes in the tissue. Therefore, importance of understanding single nucleotide variation in the mapped transcriptome is important to identify the trait-associated genomic mutations in shaping the phenotypes [7]. In this study, the pituitary gland transcriptomes of three cattle breeds: Polish HF, Polish Red, and Hereford were investigated. Polish HF and Polish Red are the native cattle breeds utilized both for dairy and beef productions, whereas the Hereford breed is used solely for beef production. It is well known that the pituitary gland transcriptome, due to its potential endocrinal role, has the major influence towards genetic improvement of economic traits, particularly the growth and reproduction trait. In general, the bovine pituitary gland is responsible for growth control at different stages of development. This involves complex interactions of several hormones and growth factors, acting in both an endocrine, and a paracrine or autocrine manner. In this context bovine growth and development in young animal ages, candidate genes such as the *pituitary growth hormone* (*GH or somatotropin*) and its receptor (*GHR*), *insulin-like growth factors* (*IGF*), *IGF binding proteins* (*IGFBP*) and the *IGF-I receptor* (*IGF-IR*), *myogenic factors* (*MYFs*), and *somatostatin*, plays an essential role in postnatal growth regulation, especially in nutrient utilization [8–11]. Generally, in cattle breeding practice and selection programs, the maximum genetic response is favorable for traits of economic importance, which includes growth traits.

In animal genomics, RNA-Seq could also provide a powerful tool for high-resolution genomic analysis of tissues and cell populations of domestic animals to identify novel mutations and transcripts affecting the major economic traits [12]. Gene expression thus is a useful marker for economic traits, suggesting that RNA-Seq could play a role in domestic animal breeding and selection programs *viz*., marker assisted selection (MAS), gene assisted selection (GAS) and genomics selection (GS) [13–15]. In animal genomics, detection of potential putative SNPs for economic traits has great potential in genetic improvement of all domestic livestock animals, including cattle. In the past decade, implementations of MAS, GAS and GS were highly recommended to the global cattle breeding program worldwide. In last 5–6 years, several studies have reported on the identification of novel SNP discoveries using RNA-seq technologies in cattle [16–25] (Table 1). To best of our knowledge, no studies have reported about the detections of SNPs in bovine pituitary gland except, some of our preliminary results presented at an international conference [26]. A brief summary of the global RNA-seq studies on the detection of SNPs in cattle is illustrated in Table 1. In this study, we used mRNA-Seq to characterize and compare the bovine pituitary transcriptomes of the Hereford, Polish Red, and Polish HF breeds, with respect to single nucleotide variations, CGs, QTLs regions and to identify the novel breed-specific SNPs.

**Table 1:** Review of literature on global tissue-specific bovine RNA-seq experiments based on SNP discoveries.

Transcriptome	Cattle breed	Economic trait	SNP discoveries	References
**Milk**	Holstein cows	Milk production trait	The SNP detection analysis revealed 100,734 SNPs in Holstein samples, and a large number of those corresponded to differences between the Holstein breed, and the Hereford bovine genome assembly Btau4.0. The number of polymorphic SNPs within Holstein cows was 33,045. The accuracy of RNA-Seq SNP discovery was tested by comparing SNPs detected in a set of 42 candidate genes expressed in milk that had been re-sequenced earlier using Sanger sequencing technology. Seventy of 86 SNPs were detected using both RNA-Seq and Sanger sequencing technologies.	[16]
**Hypothalamus of a prepubertal and a postpubertal heifer.**	Brangus (3/8 Brahman × 5/8 Angus)	Growth and fertility traits	Combined of BovineSNP50 BeadChip and RNA-seq data analysis identified SNPs from the hypothalamus of a prepubertal and a postpubertal Brangus heifer.	[17]
**Leukocytes**	Holstein, Jersey and Cholistani	Breed-specific RNA-seq experiment (None of the bovine economic traits were investigated)	Study identified breed-specific base changes in protein coding regions. Among 7,793,425 coding bases, only 165 differed between Holstein and Jersey, and 3,383 (0.04%) differed between Holstein and Cholistani, 817 (25%) of which resulted in amino acid changes in 627 genes.	[18]
**Longissimus Muscle**	Qinchuan beef cattle	Beef production	Study discovered that 30,618–31,334 putative single nucleotide polymorphisms were located in coding regions.	[19]
**blastocysts**	Bovine embryo	Fertility and reproduction trait	Study generated approximately 38 million single reads of 40 bp per embryo, with an average of approximately 29 million reads per sample(76.9%) mapped to annotated portions of the Ensembl-UMD3.1 genome. Expressed biallelic SNP variants with allelic imbalances were observed in 473 SNP, where one allele represented between 65–95% of a variant’s transcripts.	[20]
**Horn Cancer (HC) and Horn Normal (HN)**	Kankrej	Complex disease trait (cancer)	Study found 9532 and 7065 SNPs as well as 1771 and 1172 Indels in HC and HN, respectively, from which, 7889 SNPs and 1736 Indels were uniquely present in HC, 5886 SNPs and 1146 Indels were uniquely present in HN and reported first time in Bos indicus.	[21]
**Longissimus thoraci**	Limousine bulls	Meat quality trait	For the meat quality trait 34,376 different SNPs were detected. Fifty-five percent of the SNPs were found in coding regions, and ~22% resulted in an amino acid change. Applying a very stringent SNP quality threshold, we detected 8,407 different high-confidence SNPs, 18% of which are non-synonymous coding SNPs.	[22]
**Hypothalamus, pituitary gland, ovary, uterus, endometrium, longissimus dorsi muscle, adipose, liver**	Brangus heifers, Brahman (Bos indicus) x Angus (Bos Taurus)	Fertility traits: age of first observed corpus luteum (ACL), first service conception (FSC), and heifer pregnancy (HPG)	Study revealed 25 QTL loci containing SNPs associated with fertility trait.	[23]
**Subcutaneous adipose tissue (fat) of a fetal, adult bulls, adult heifers and adult steers**	Qinchuan	Beef production	Study detected 56,564 (fetal), 65,154 (adult bull), 78,061 (adult heifer), and 86,965 (adult steer) putative SNPs located in coding regions of the four pooled libraries of adipose tissue.	[24]
**Mycobacterium avium subspecies paratuberculosis (MAP)**	German Holstein cattle	Health disease trait	The genetic variability within a pool of seven genes (*LAMB1*, *DLD*, *WNT2*, *PRDM1*, *SOCS5*, *PTGER4*, and *IL10*) were investigate in a population of 324 German Holstein cattle (162 cases MAP positive and 162 controls MAP negative) by GWAS/RNA-Seq analysis. Study discovered novel SNPs putatively associated with paratuberculosis susceptibility.	[25]

## Results

### mRNA sequencing and read alignment

To obtain the bovine pituitary gland transcriptome at a single-nucleotide resolution, two biological replicates of poly(A)-enriched mRNA of young bulls aged 6, 9, and 12 months from three cattle breeds were converted to barcoded strand-specific dUTP RNA-seq libraries, and sequenced on the Illumina NextSeq 500 sequencer. Sequencing generated a total of 113,882,3098 raw paired-end reads with a length of 156 bases. The reads were de-multiplexed to assign reads to each sequenced sample according to its index. An average of 63 million paired-end reads was obtained per library (n = 18). The FastQ sequence dataset of each library was submitted to NCBI-SRA experiment number SRS1296732 (http://www.ncbi.nlm.nih.gov/sra?linkname=bioproject_sra_all&from_uid=312148). Using the BWA programme, breed-specific pituitary gland transcripts were mapped to the bovine reference genome (UMD3.1 assembly plus Y chromosome) and the results were summarized in Table 2 (Polish HF), Table 3 (Polish Red) and Table 4 (Hereford), respectively. Based on the BWA mapping programme under default condition, 93.04%, 94.39% and 83.46% of the mapped sequencing reads were properly paired for the Polish HF, Polish Red, and Hereford breeds, respectively (Tables 2–4).

**Table 2:** Transcriptome mapping of pituitary gland in Polish HF breed to bovine reference UMD3.1 genome assembly.

Age	Total	Mapped	Paired in sequencing	Read1	Read2	Properly paired	With itself and mate mapped	Singletons	Mate mapped to a different chromosome
	1x10^6^	1x10^6^	1x10^6^	1x10^6^	1x10^6^	1x10^6^	1x10^6^	1x10^6^	1x10^6^
**6m**	218,45	218,45	109,26	109,19	173,99	202,27	4,34	8,13	1,89
**9m**	159,83	159,83	79,84	79,98	132,45	148,29	2,38	3,17	0,59
**12m**	137,10	137,10	68,58	68,52	118.33	128,94	1,77	2,07	0,48
**Total**	515,38	515,38	257,38	257,70	424,78	479,51	8,49	13,38	2,97

**Table 3:** Transcriptome mapping of pituitary gland in Polish Red breed to bovine reference UMD3.1 genome assembly.

Age	Total	Mapped	Paired in sequencing	Read1	Read2	Properly paired	With itself and mate mapped	Singletons	Mate mapped to a different chromosome
	1x10^6^	1x10^6^	1x10^6^	1x10^6^	1x10^6^	1x10^6^	1x10^6^	1x10^6^	1x10^6^
**6m**	108,49	108,49	54,27	54,22	94,08	102,68	1,36	1,14	628,78
**9m**	56,89	56,89	28,36	28,53	48,48	54,14	0,70	0,69	329,58
**12m**	50,00	50,00	25,03	24,96	42,02	46,48	0,71	0,78	287,20
**Total**	215,39	215,39	107,66	107,72	184,60	203,31	2,78	2,62	1 245,58

**Table 4:** Transcriptome mapping of pituitary gland in Hereford breed to bovine reference UMD3.1 genome assembly.

Age	Total	Mapped	Paired in sequencing	Read1	Read2	Properly paired	With itself and mate mapped	Singletons	Mate mapped to a different chromosome
	1x10^6^	1x10^6^	1x10^6^	1x10^6^	1x10^6^	1x10^6^	1x10^6^	1x10^6^	1x10^6^
**6m**	207,07	202,72	207,07	102,64	104,43	174,57	200,69	2,03	6,30
**9m**	105,39	103,66	105,39	52,01	53,37	82,16	102,80	0,86	6,31
**12m**	95,58	94,07	95,59	47,41	48,17	78,77	93,30	0,78	2,94
**Total**	408,04	401,97	408,04	202,06	205,98	335,51	396,79	3,67	15,54

### SNP discoveries in cattle breeds

**Fig 1:**
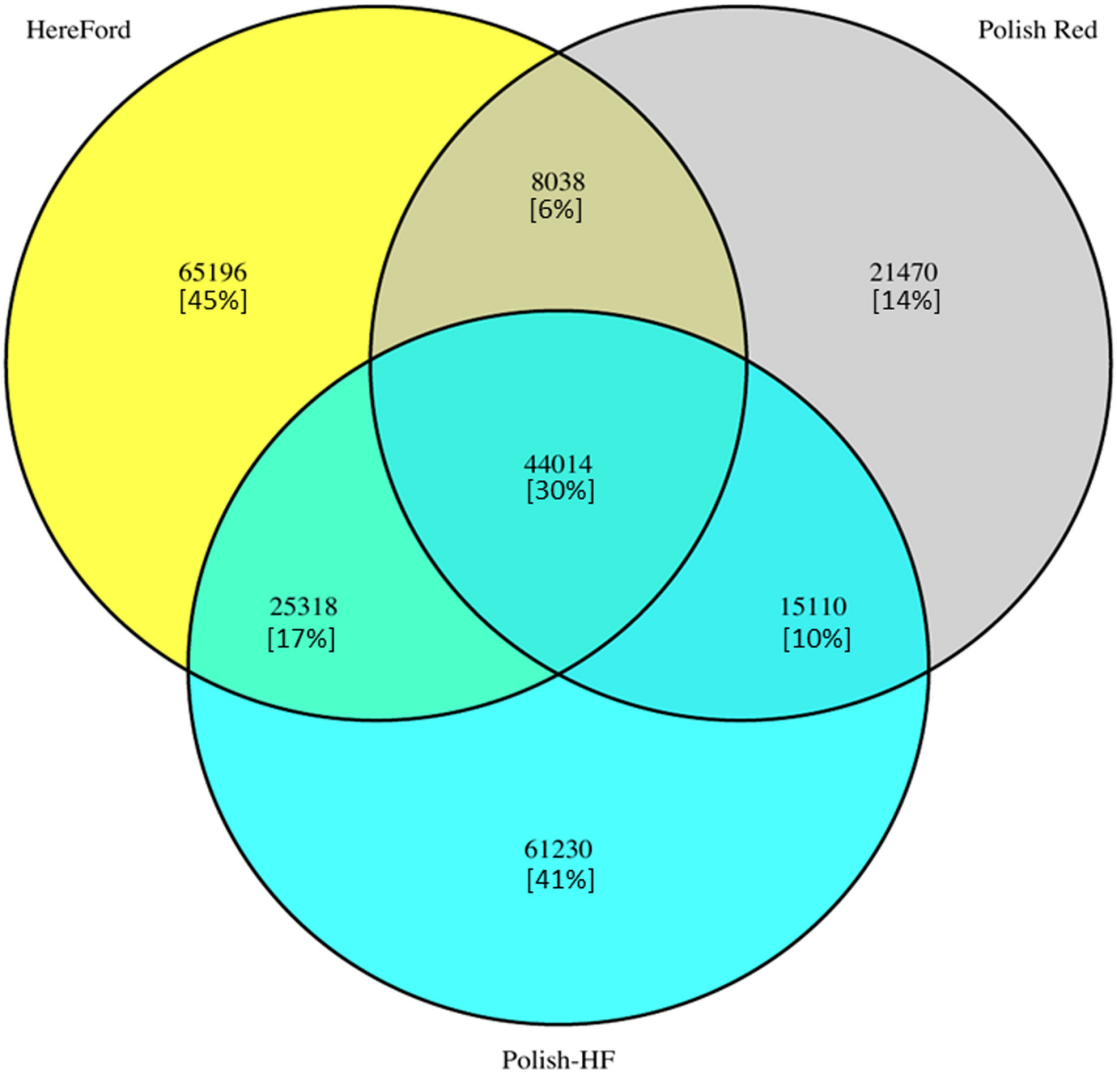
Venn diagram showing the number of SNP segregating within three cattle breeds.

Using a stringent filtering parameter of read count (SAMtools package), with a minimum depth of 5, detection of putative SNPs were performed in three breeds. The Venn diagram is displayed to report the number of SNPs shared among three cattle breeds (Fig 1). For the detection of breed-specific putative SNPs, we utilized two stringent parameters, *viz*., a minimum 10 SNP reads per base with an accuracy ≥ 90%, and a minimum 10 SNP reads per base with an accuracy = 100%. The SAMtools identified single base substitutions (SNPs) as well as small insertions and deletions (*indel*); however, only SNPs results were considered to publish in this paper. A total of 13.7 million breed-specific SNPs positions were detected with the RNA-Seq reads, with an average of 765,326 (~ 0.76 million) SNPs per young bull (S1–S18 Tables). This high percentage of novel SNPs suggests that a large fraction of the genetic variability present in cattle breeds still remains to be discovered. Furthermore, based on the S1–S18 Tables, the raw SNP database (SNP-db) of pituitary gland transcriptome representing the investigated young bulls of the Polish HF, Polish Red, and Hereford breeds was constructed (S19 Table). Using two stringent filtering parameters, SNP-db records were trimmed to construct a highly reliable SNP-db and resulting in reduction of 95,7% and 96,4% cut-off mark of constructed raw SNP-db (S19 Table).

Breed-specific trimmed/filtered SNP-db showed the highest SNP counts in the Polish HF breeds with an average (per young bull) 880,442 raw SNPs, 39,730 SNPs (≥10reads ≥90%) and 31,978 SNPs (≥10reads = 100%), followed by 754,719 raw SNPs, 34,658 SNPs (≥10reads ≥90%), 30,052 SNPs ≥10reads = 100%) in Hereford breed, and 660,819 raw SNPs, 25,414 SNPs (≥10reads ≥90%), 20,573 SNPs (≥10reads = 100%) for Polish Red breed, respectively. Furthermore, the SNP-db showed the highest percentage of ≥10reads ≥90% and ≥10reads = 100% filtered SNPs in Hereford breed (4.6% and 4.0%), followed by Polish HF breed (4.5% and 3.6%), and Polish Red breed (3.8% and 3.1%), respectively.

Based on their types and occurrences, filtered SNP-dbs [S20 and S21 Tables] were further categorized into the following four sub-categories, as shown in S22 Table.

1. SNPs represented in any of six samples per given breed (SNP-type designated as ANY)
2. SNP detected in all six samples per breed (SNP-type designated as Breed-specific).
3. SNP detected in any of six samples per given breed, not representing any other breed (SNP-type designated as ANY UNIQUE)
4. SNP detected in all six samples per breed, not representing any other breed (SNP-type designated as Breed-specific UNIQUE).

The S22 Table showed that SNP categorization substantially reduced the SNP-db, resulting in highly reliable breed-specific, and breed-specific unique SNP-dbs. Moreover, the breed-specific UNIQUE SNP-db were further explored and illustrated into three S Tables for the Hereford (S23 Table), Polish Red (S24 Table), and Polish HF breeds (S25 Table), respectively. Finally, Eight putative SNPs from Polish HF were selected from one of the unique SNP-db (S25 Table), for SNP**s** validation experiment.

In the following sub-section of SNP**s** analysis, it was assumed that certain regions of *Bos taurus* genome are still unknown (base = N, according to recent mapping UMD3.1). After applying the both SNP filtering criteria (≥10 SNP reads with an accuracy ≥90% and = 100%), a total of 1039 best candidates of *de novo* SNPs reads were identified. (S26 and S27 Tables). Lastly, it was also assumed that certain regions of the reference *Bos taurus* genome still contained mutations or errors which were not correctly annotated. Such errors were corrected by analysing the SNP**s** database through successive annotations. After applying the filtering criteria of ≥10 SNP read**s** with an accuracy ≥90%, a total of 5.041 potential putative SNPs were identified, whereas**,** filtering criteria of ≥10 SNP read**s** with an accuracy of = 100%, a total of 567 potential putative SNPs was identified (S28 Table).

#### Breed-specific SNPs discoveries and QTLs/CGs analysis

**Fig 2:**
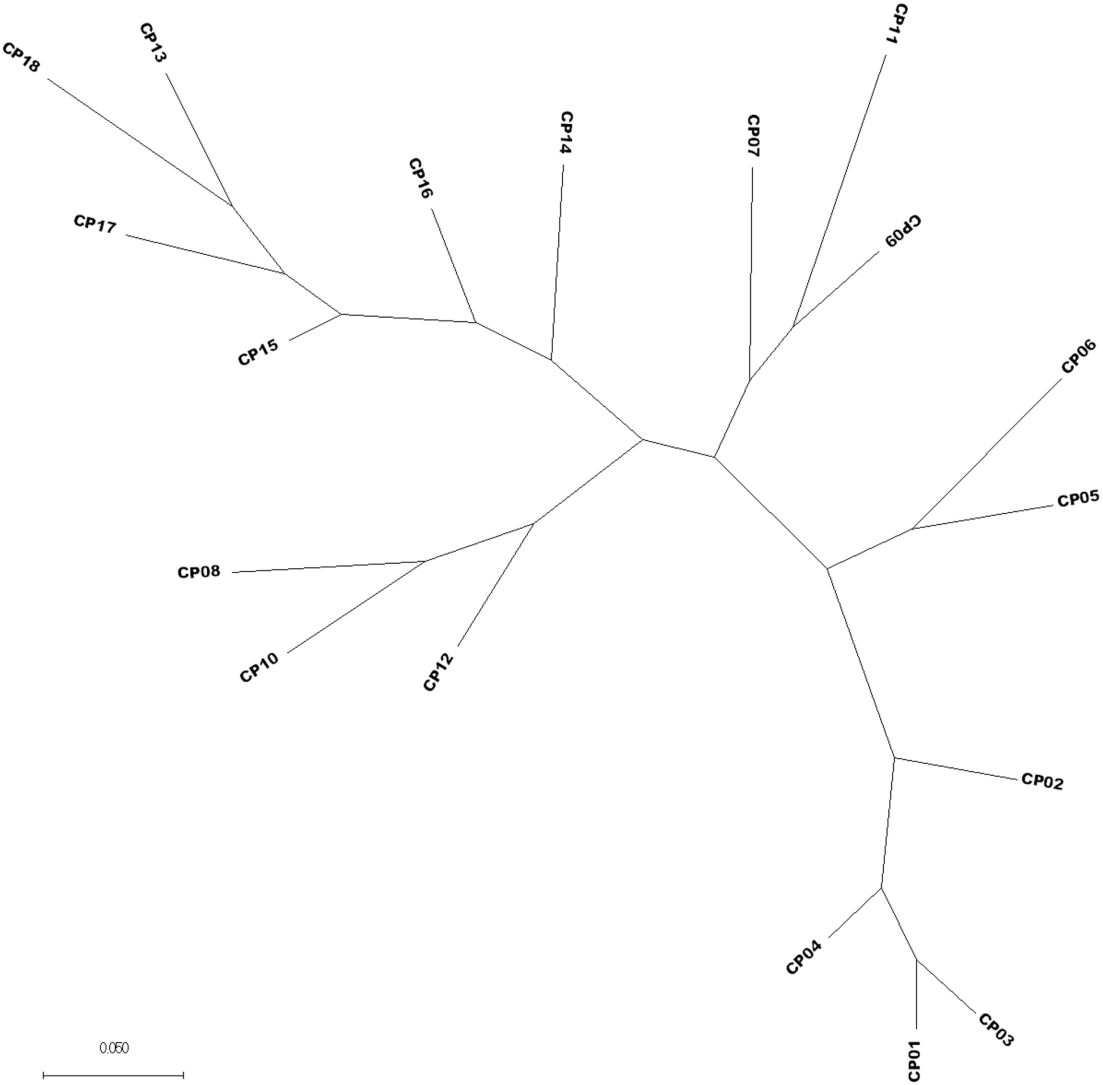
The phylogenetic relationship among RNA-seq samples using Maximum likelihood method. An unrooted phylogeny tree of 18 bulls’ samples representing CP01-CP06 (Hereford), CP07-CP12 (Polish Red) and CP13-CP18 (Polish-HF). All nodes were robust at 100% bootstrap support. The scale bar denotes substitutions per site**.**

For each breed, a set of 76 QTLs/CGs loci (http://www.animalgenome.org/cgi-bin/QTLdb/index) was comprehensively investigated (S29 Table). The chromosomal locations and SNP locations of identified putative SNPs loci for each breed representing three developmental ages were summarized in detail in supplement S30–S32 Tables respectively. This comprehensive QTLs/CGs analysis, allowed us to identify within breed and between breeds putative SNPs for each breed and developmental ages of young bulls (S33–S35 Tables). The results showed that most of the identified top 20 CGs were represented in all three breeds and developmental ages, however, numbers of SNPs hits loci varied across developmental ages. Furthermore, *KCNIP4*, *CCSER1*, *DPP6*, *MAP3K5 and GHR* genes loci were identified as top 5 potential CGs with highest SNPs hit loci in all three breeds and developmental ages, while *CAST* gene locus was identified as potential CGs expressed with more than 100 SNPs hit in Polish HF and Hereford breeds (S30–S35 Tables). Additionally, a phylogenetic tree [27] was constructed to see how similar the cattle within the same breed. Results revealed that all three breeds were clustered together for each breed and were separated from for each other (Fig 2). Thus, the study proved to indicate that SNP calling form RNA-seq data has been effective at identified genetic variation.

### Breed-specific SNP Validation

**Fig 3:**
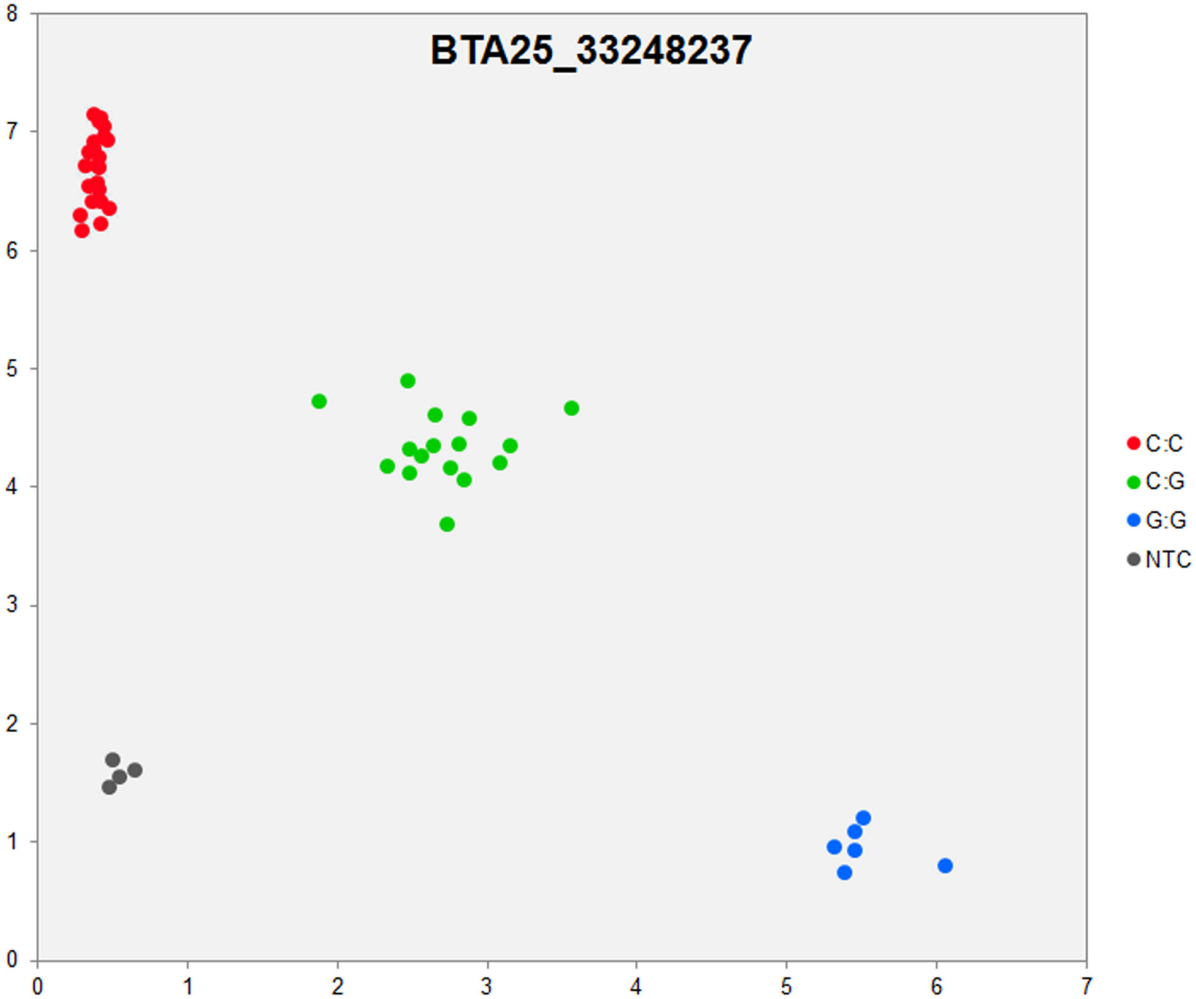
KASP SNP genotyping assay of BTA25_ 33248237 locus showing the data for single KASP assays on a single cluster plot.

**Fig 4:**
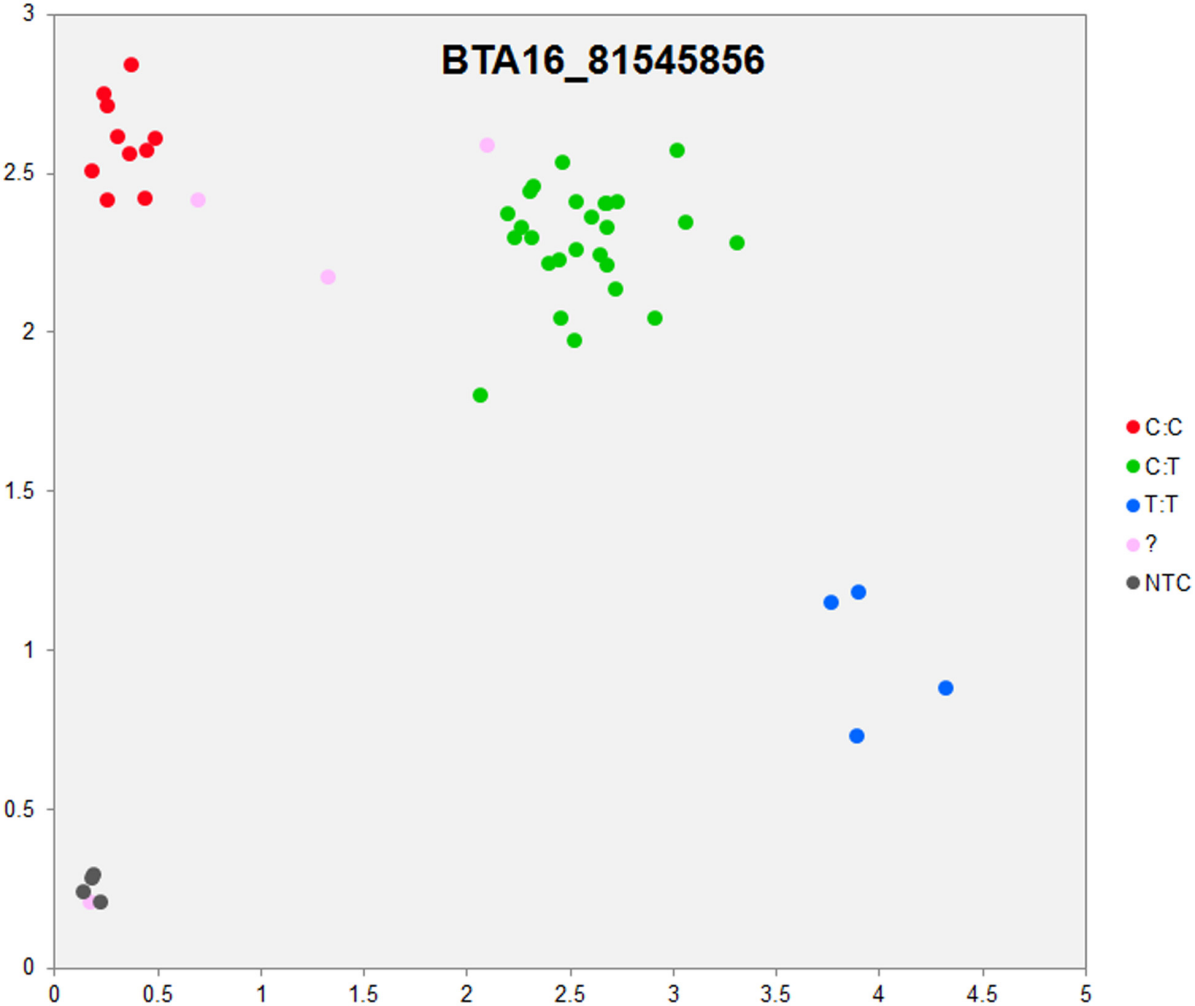
KASP SNP genotyping assay of BTA16_ 81545856 locus showing the data for single KASP assays on a single cluster plot.

**Fig 5:**
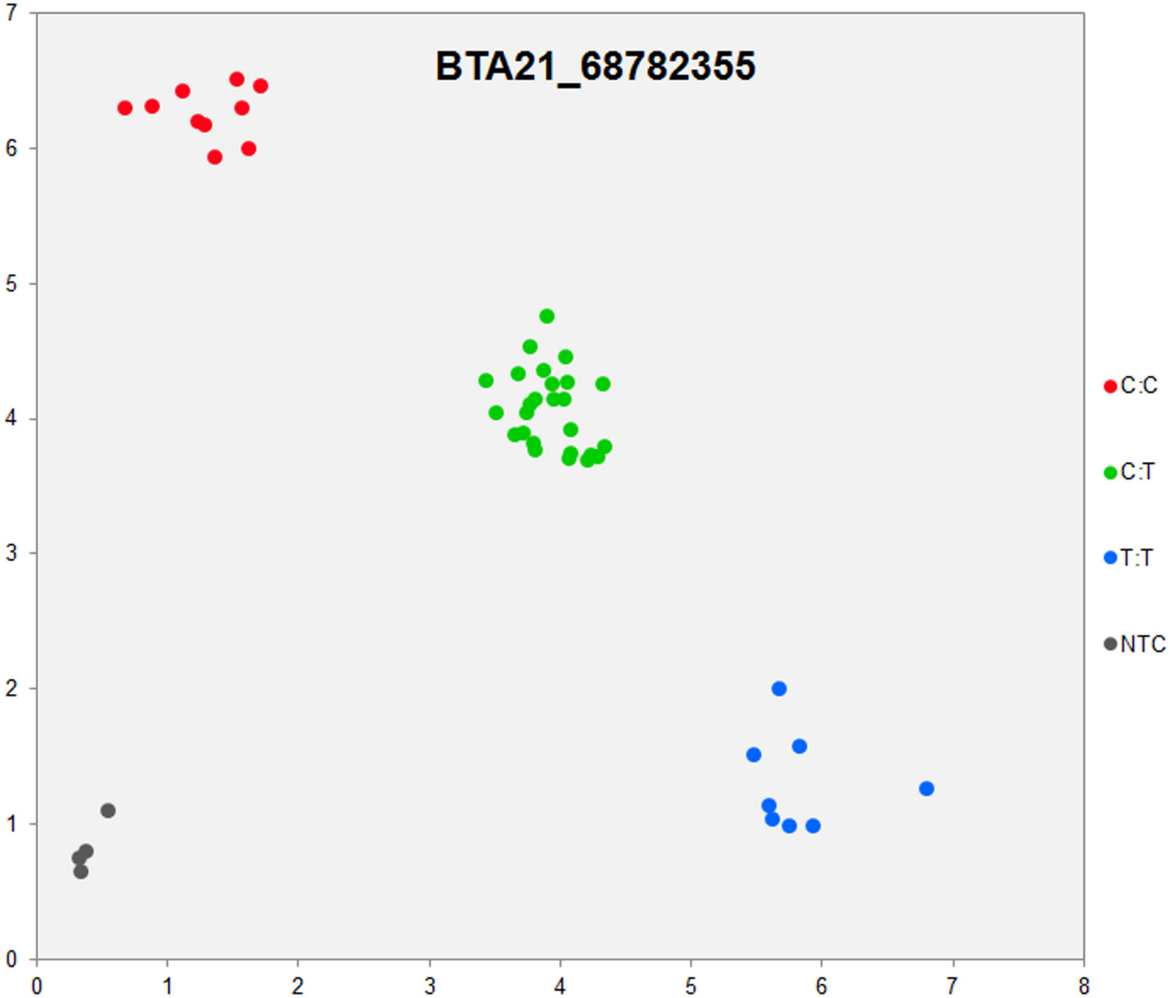
KASP SNP genotyping assay of BTA21_ 68782355 locus showing the data for single KASP assays on a single cluster plot.

**Fig 6:**
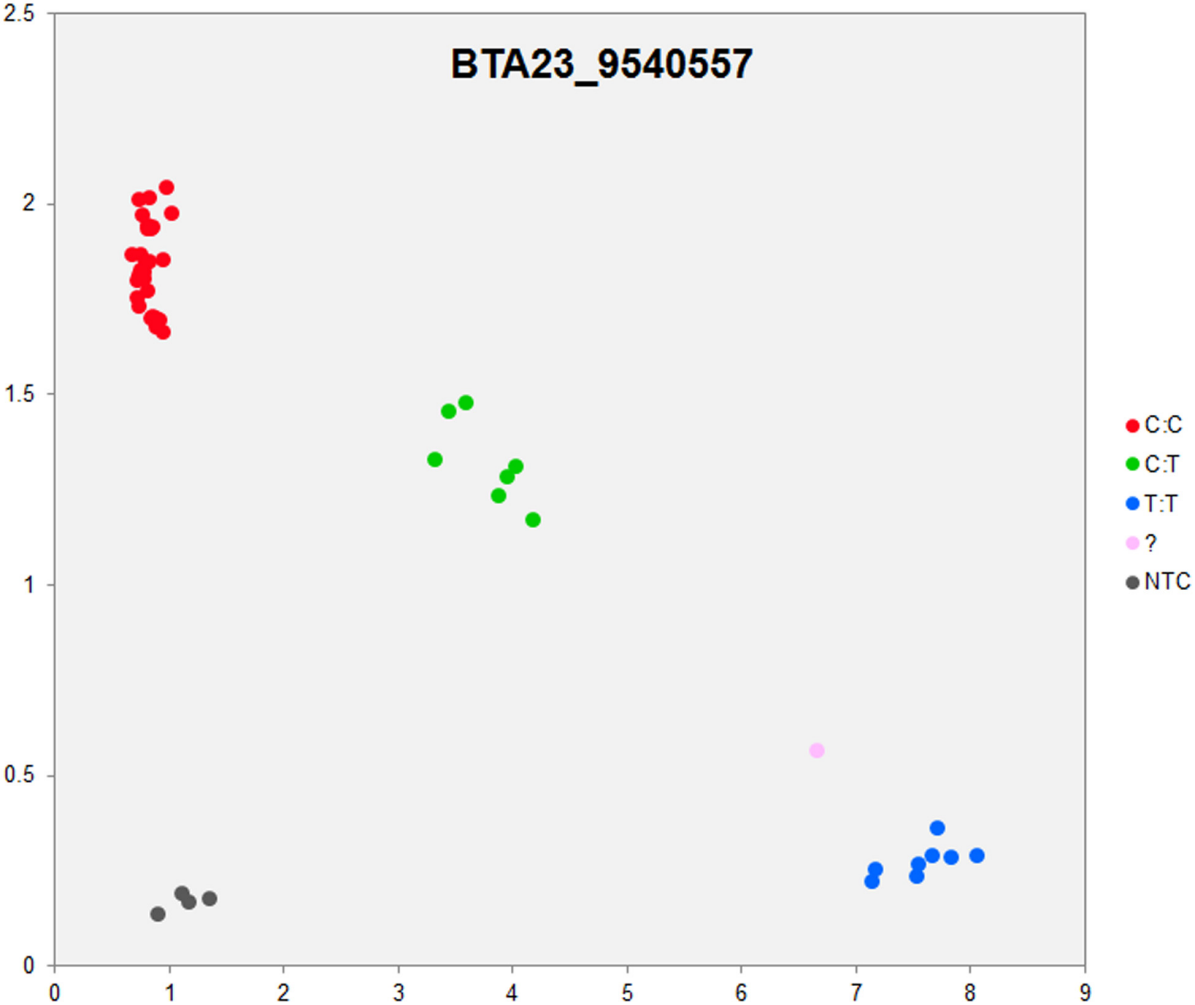
KASP SNP genotyping assay of BTA23_ 9540557 locus showing the data for single KASP assays on a single cluster plot.

**Fig 7:**
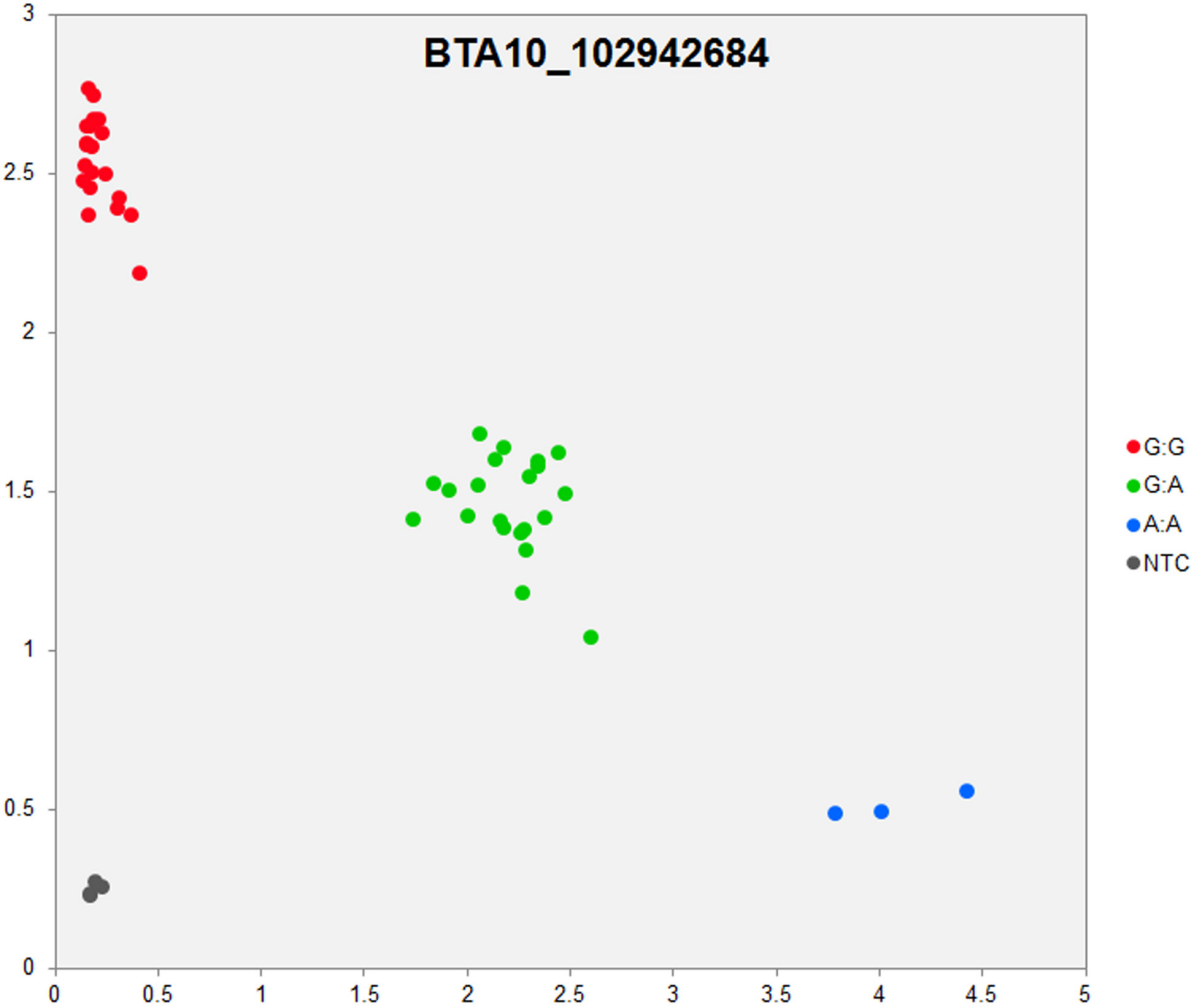
KASP SNP genotyping assay of BTA10_ 102942684 locus showing the data for single KASP assays on a single cluster plot.

**Fig 8:**
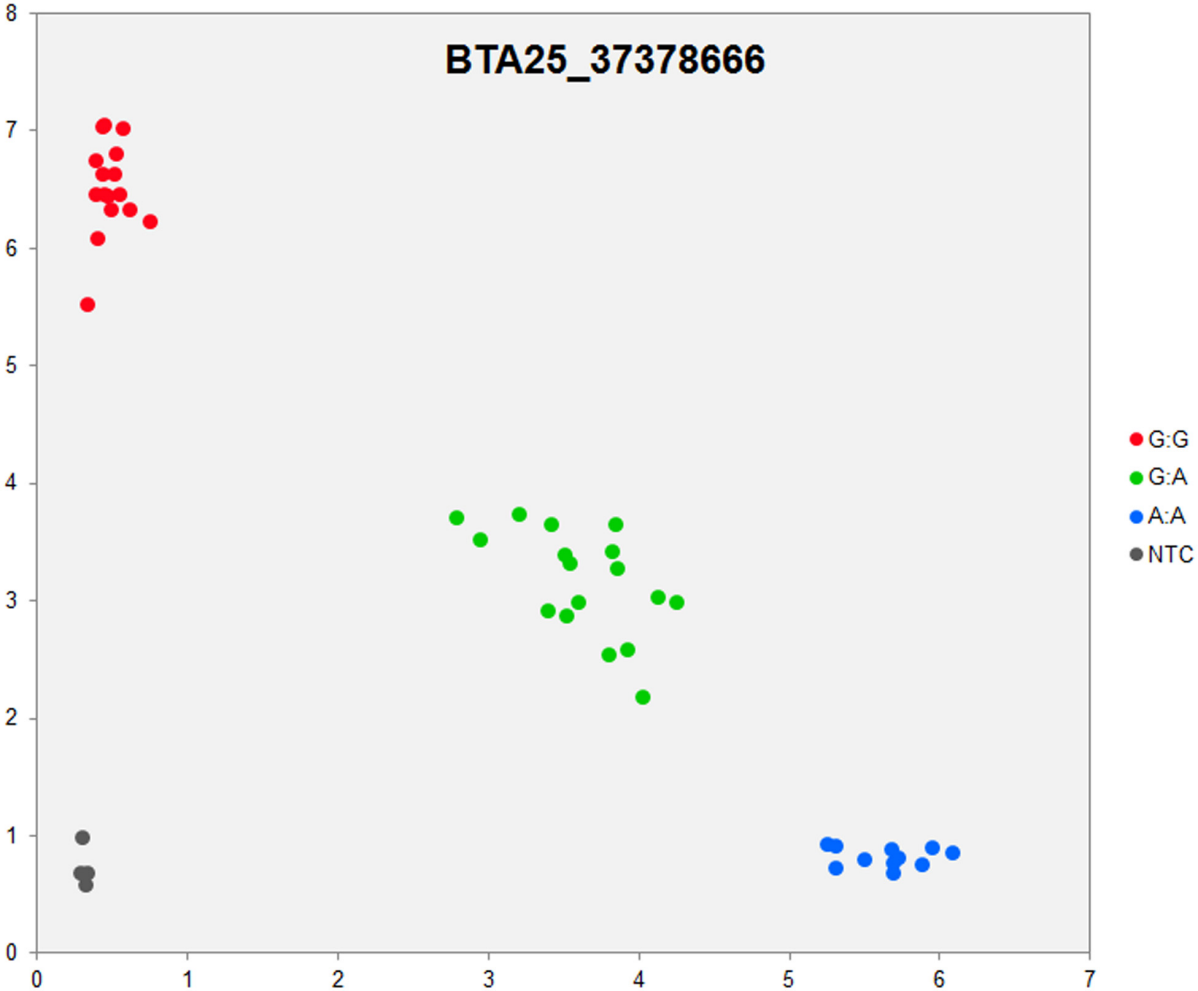
KASP SNP genotyping assay of BTA25_ 37378666 locus showing the data for single KASP assays on a single cluster plot.

**Fig 9:**
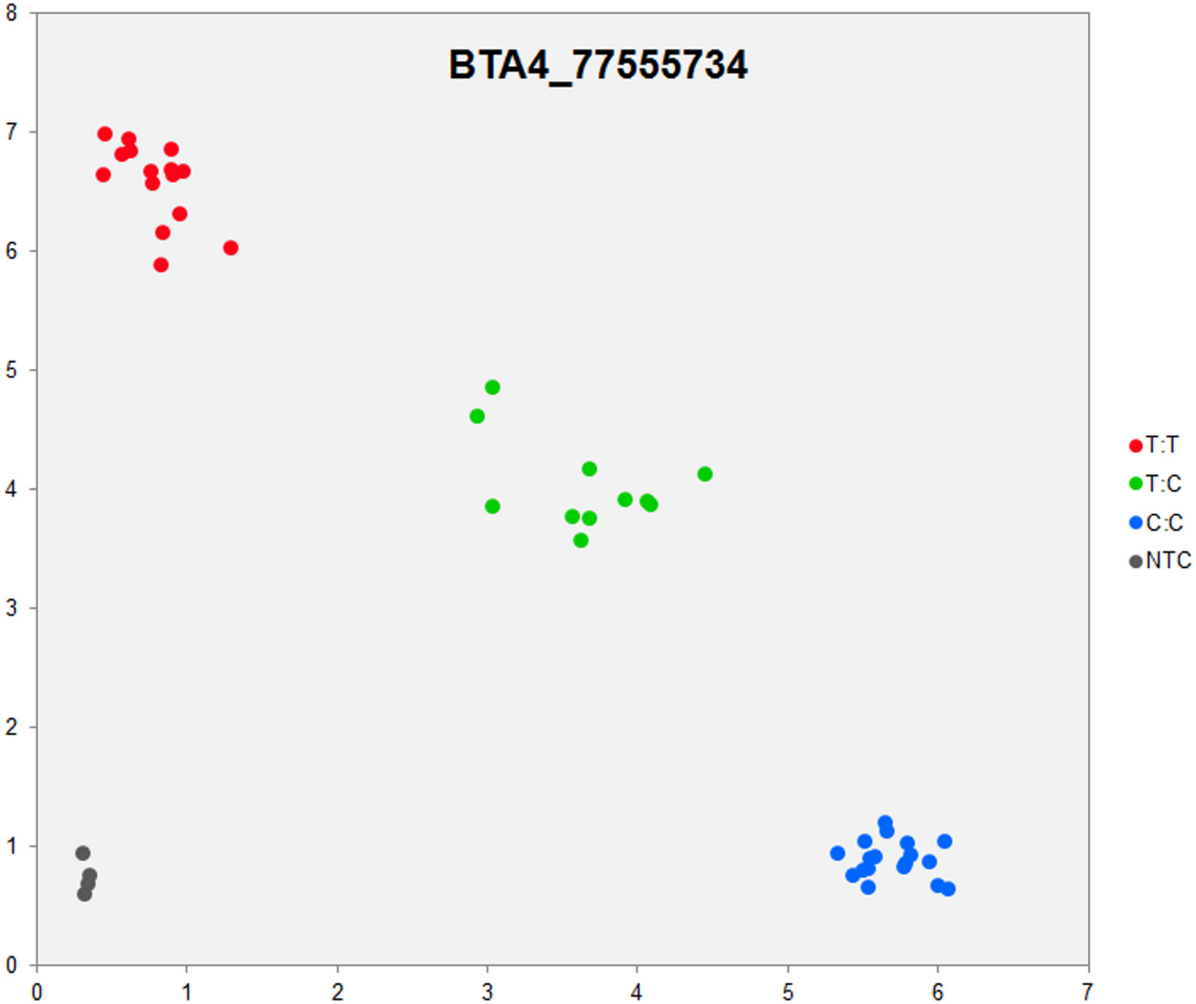
KASP SNP genotyping assay of BTA4_ 77555734 locus showing the data for single KASP assays on a single cluster plot.

**Fig 10:**
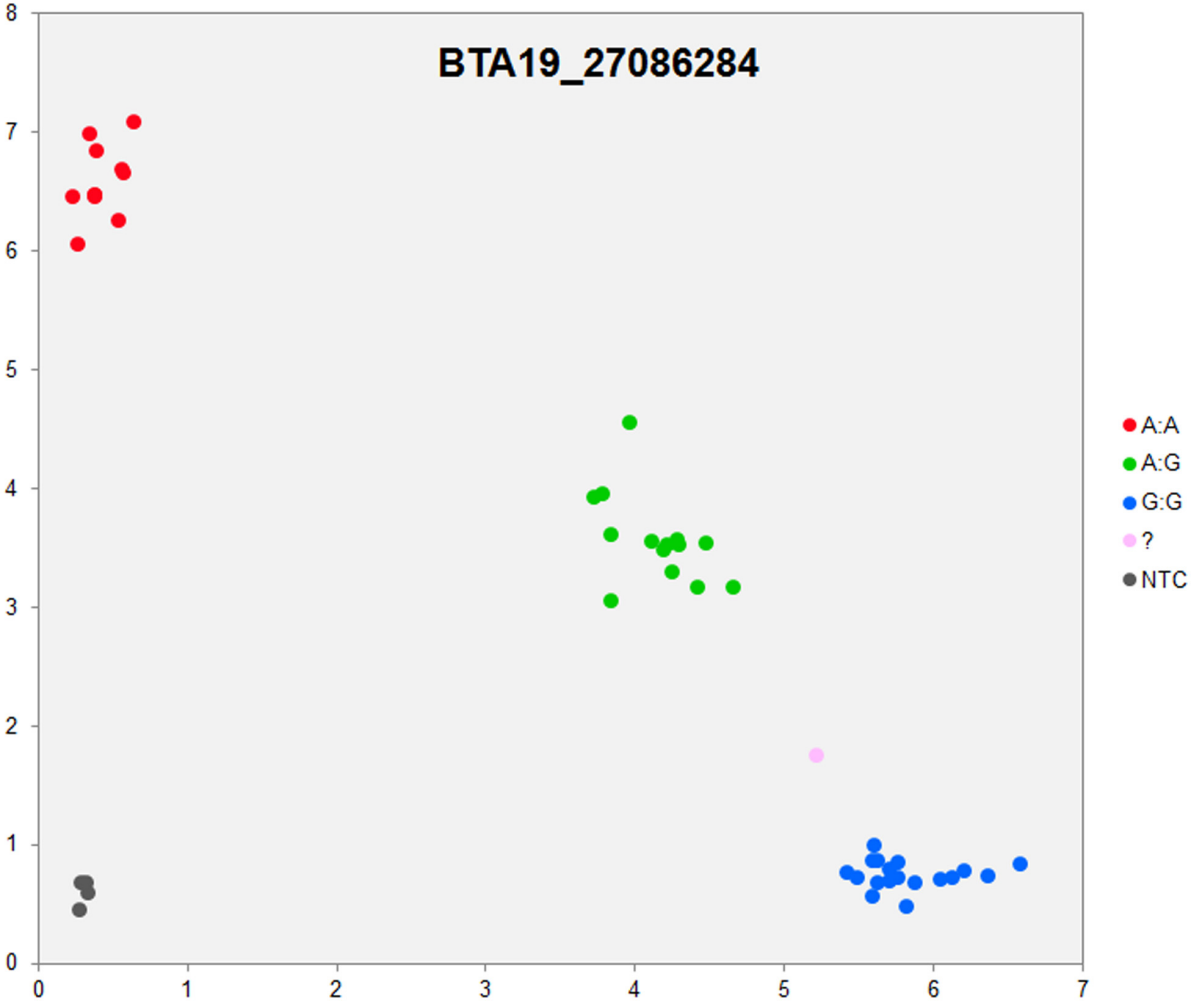
KASP SNP genotyping assay of BTA19_ 27086284 locus showing the data for single KASP assays on a single cluster plot.

In order to validate the breed-specific SNP discovery process, a subset of eight putative SNPs**,** identified as uniquely specific to the Polish HF breed**,** were evaluated using single-plex KASP^™^ assays (LGC Genomics), which are based on fluorescently labelled allele-specific PCR primers. The SNP identity with normal and mutant alleles, genome locations, SNP gene descriptions of selected SNPs are presented in S36 Table. An additional analysis for the estimation of direct relationship between breed and detected SNP polymorphisms was performed using the PROC MIXED SAS 9.2 package with the age as a random effect. Results revealed no significant association between developmental ages and SNPs selected for the validation. However, significant and highly association between breed and SNPs selected for validation were observed (S37 Table). Based on this initial analysis, KASP^™^ SNP assay results were organized according to distributions of genotypes for each validated SNP**s** locus in all the investigated young bulls from three breeds (S38 Table). Furthermore, the distribution of and frequencies of SNP**s** alleles (S39 Table) results revealed the highest numbers and frequencies of mutant alleles in the SNP loci of all young bulls from the three breeds (n = 44), except, BTA4_77555734 and BTA19_27086284 loci. Moreover, the genotypes distributions of each validated SNP were further illustrated specifically for each breed, and are presented in S40 Table. Based on the observed genotypes and allele frequencies (S38–S40 Tables), the results revealed that the selected SNPs mutations were polymorphic to tested cattle breed population, and were in accordance with RNA-seq observations. Furthermore, a subset of eight breed-specific putative SNP makers from the Polish HF breed worked well to KASP^™^ SNP genotyping assay (LGC Genomics), and did not reveal either non-amplification or ambiguous clustering, except in few samples which did not respond to KASP^™^ assay because of poor DNA quality (Figs 3–10).

Based on the initial KASP^™^ SNP assays results (S38–S40 Tables), the statistical analysis of SNP validation was carried out by the Genepop software [28, 29]. Using the Fisher's Exact Probability test, the genetic differentiation of SNP alleles and SNP genotypes results revealed significant differences in the SNP alleles frequencies (S41 Table), and SNP genotypes frequencies (S42 Table) among the investigated cattle breeds and SNP loci. Moreover, the comparison of allele and genotypic frequencies of selected SNPs among the investigated cattle breeds showed that highly significant differences between the Polish HF breed vs Polish Red breed, and the Polish HF breed vs Hereford breed, thus confirming the validation of selected Polish HF breed-specific SNP markers. Moreover, using a Markov chain method the SNP validations results were further confirmed by testing the deviation from Hardy Weinberg equilibrium (HWE) for each SNP locus (S43 Table), and each breed population (S44 Table). The results revealed highly significant differences in SNP genotypes and alleles frequencies among the cattle breeds across all eight SNP loci and all three breeds.

## Discussion

In cattle genomics, the key challenge in SNP discovery is to distinguish true individual variants (occurring at a low to very low frequencies) from sequencing errors (which often occurs at higher frequencies). Breed-specific SNP discovery is critical for successful implementation of GAS and GS programs for genetic**s** improvement in economically important traits [10–12]. With increasing attention being paid to genomic selection based on the next generation sequencing (NGS) by animal breeders, the primary goal of genome-wide SNP discovery using NGS is to identify large number of markers that provide complete set of genetic variants for economic traits. Herein this paper by using the NGS based RNA-seq strategy, the pituitary gland transcriptome analysis of the three cattle breeds was accomplished by performing the alignment of mapping read, followed by detections of breed-specific SNPs, and finally the validation of SNPs.

### Alignment of mapping reads

The mapping of sequence reads was performed with BWA (STAR alignment tool) using their default settings on the same Linux based servers. The study of Wang and colleagues suggested both Bowtie and BWA tools could be utilized for fast and efficient alignment of Illumina short reads [30]. However, other study [31] found that BWA was superior to Bowtie at mapping short reads. For example, a higher percentage of read mappings were achieved using BWA (84.8%) compared to Bowtie (62.3% including the suppressed reads) when Illumina reads of eight genotypes were mapped onto the reference sequence. Therefore, in this paper, the BWA based assembly was chosen for downstream analysis because it produced higher percentages of mapped reads.

In cattle genomics, very few tissue-specific RNA-seq experiments on SNP discoveries were reported. According to the PubMed literatures, no RNA-seq experiment on SNP discoveries in bovine pituitary gland transcriptome has been conducted so far, besides our preliminary results [26]. A previous study on the longissimus thoraci transcriptome from three Limousine bull calves revealed approximately 36–46 million paired-end reads per library, and 63% to 67% of the mapped reads were aligned properly to paired end with almost negligible transcriptome contamination, using the BWA program [22].

By sequencing the milk transcriptome mapped uniquely onto the bovine genome, approximately 65% of the RNA-Seq reads were successfully mapped and aligned to the reference genome, and approximately 17,000–19,000 genes were expressed in milk [32]. In an another bovine RNA-seq experiment on rumen epithelium tissue, the study of Baldwin and co-workers found that ~71% of the reads were mapped onto ~17,000 different genes of *Bos taurus* reference genome [33].

A previous study of RNA-seq study on bovine milk transcriptome identified 118 million reads of 36 to 40 bp size, averaging 17 million short-sequence reads for each milk sample. Study further revealed that approximately 70% (82.7 million) reads were properly mapped and remaining 35 million were unmapped. Approximately 87.5% of uniquely mapped reads corresponded to total exon reads, and a small remaining fraction corresponded to total intron reads [16].

While analysing the bovine embryo, the RNA-seq experiment on single bovine blastocysts reported approximately 38 million sequencing reads per embryo, and 9,489 known bovine genes expressed with high correlations of expression levels between the samples. [20]. In another study using multiple tissues (*i*.*e*., hypothalamus, pituitary gland, ovary, uterus, and endometrium, longissimus dorsi muscle, adipose, and liver) identified an average of 30 million sequences reads from each sample tissue, and mapping of approximately 70 to 80% of the sequence reads to the 27,368 annotated genes in the bovine genome assembly UMD3.1.74 [23].

While analysing the RNA-seq experiment of bovine leukocyte transcriptomes from two taurine breeds (Holstein and Jersey), and one indicine breed (Cholistani), a total of approximately 21 million paired-end fragments were sequenced for each of the three breeds. The Tophat results revealed that an approximately 70% of the sequenced fragments were successfully aligned. The study further concluded that more than 90% aligned fragments were mapped to unique genomic regions [18].

Recently, the Qinchuan beef cattle transcriptome analysis in context to longissimus muscle growth and development identified approximately 2 billion pair-end reads of 100 bp in length for bovine longissimus muscle from three embryos and three adult bovines. For Emb135 and 30M samples, approximately 79% and 77% pair-end reads were mapped to the UMD 3.1 reference genome [19].

### Breed-specific SNP discoveries

The alignment file (.gz and.txt format) generated by BWA was used as input files for bovine pituitary gland SNP discovery using SAMtools [34]. The SNP detections were performed using two different stringent parameters of SNP filtering process approaches. The overall constructed SNP database for three breeds yielded approx. 13.7 million of SNPs. While, after filtering to different stringent parameters, 0.59 million and 0.49 million SNPs were detected for the stringent parameters of with minimum 10 SNP reads per base and with an accuracy of ≥ 90% and = 100%, respectively.

In an analysis of the transcriptome, utilizing RNA-seq of bovine Longissimus thoraci from three Limousine bull calves a total of 34,376 different SNP were detected [22]. Amongst these SNPs, 8,974 (26%) were homozygous in all three sequenced samples, corresponding presumably to differences between Limousine and Hereford. This study also found that there were 30,998 bi-allelic SNPs mapping to coding regions, 38.6% of which were previously found and recorded in dBs [22].

In a study on milk transcriptome, approximately 100,000 SNPs located in genes expressed in milk samples from the Holstein cows were identified [16]. However, only 32% of SNPs (33,045) were polymorphic within their seven Holstein cows. In another study, [23], putative SNPs within three investigated cattle breeds *i*.*e*., the Holstein, Jersey, and Cholistani were reported. Results showed that among 22,100,344 surveyed bases in Holstein, 32,547 (0.147%) were reliably called SNPs, and 39,370 out of 19,967,581 (0.197%) bases were polymorphic within Cholistani. Using different SNPs variant calling tools, a total of 31,334 and 30,618 putative SNPs were identified for the longissimus pooled samples of adults and pooled samples of embryo. For both samples, the SNP detection stringency conditions of i) at least two unique mapping reads that support the polymorphic nucleotide, and ii) a quality score of ≥20 were utilized [19].

The potential applications of RNA-Seq present unique benefits in terms of SNP analysis because of its wide dynamic range and ability to identify functional sequence variants. The detection and categorization of SNPs within animal production systems have been performed extensively [15]. The use of these genetic variants as markers and predictors of performance in a large variety of economic traits (*i*.*e*. meat production, milk production, fertility, calving ease, etc.) is quite common in animal breeding practices [35–37]. In the context of early body growth and development trait, classifying SNPs between samples of varying viability, ages, or breed allows for discovery of novel markers of growth traits and characterization of critical regulatory mechanisms of body growth and development. Traditionally, the use of transcript sequence for SNP variant detection has been performed with various assays [38], and more recently in cattle using RNA sequencing data [16].

## Conclusions

This is the first study to report breed-specific SNPs discovery using NGS based RNA-seq in bovine pituitary gland. The study generated nearly fourteen million breed-specific SNP-db based on the expressed genes of the bovine pituitary gland, with mapping accuracy comparable or better to previous works in *Bos taurus*. These databases of breed-specific SNP as well as identified putative SNPs within the QTLs/CGs for bovine body growth and development trait will improve the genomic resources available for cattle, especially for beef breeds, and may also prove useful to study the mechanisms underlying the genetic variability in meat quality and reproduction traits. KASP^™^ SNP assay was proven to be an efficient cost effective method to validate the breed specific putative SNPs originating from RNA-seq experiments.

## Materials and Methods

### Animals

The bovine pituitary gland samples from young growing bulls were collected from Institute of Genetics and Animal Breeding, Polish Academy of Science (PAS), Jastrzębiec, Poland. After slaughtering of the animals, the collected tissues were immediately kept in liquid nitrogen, and finally stored in deep freezer at –80°C. All procedures involving animals were performed in accordance with the guiding principles for the care and use of research animals, and were approved by the local ethics commission (permission No. 3/2005) of Institute of Genetics and Animal Breeding, Polish Academy of Science (PAS), Jastrzębiec, Poland.

The experimental design comprised of 18 young bulls for RNA-seq experiment, and 44 young growing bulls for SNP validation experiment (Table 5), in a panel of three selected cattle breeds: Polish HF, Polish Red, and Hereford, respectively. All experimental animals were reared at Institute of Genetics and Animal Breeding, Jastrzębiec, Poland in a closed herd, and providing uniform feeding and environmental conditions.

**Table 5:** The distribution (n) of investigating young bulls in a breed-specific experimental design representing bovine pituitary gland transcriptome.

Breeds	6 months	9 months	12 months	Total
**RNA-seq experiment**				
**Polish HF**	2	2	2	6
**Polish Red**	2	2	2	6
**Total**	6	6	6	18
**SNP validation experiment**				
**Hereford**	2	2	2	6
**Polish HF**	2	2	2	6
**Polish Red**	2	2	2	6
**Total**	6	6	6	18

### Laboratory procedures

Total RNA was extracted and prepared from 50–60 mg of frozen bovine pituitary gland tissues (n = 18) using the guanidinium thiocyanate method [39] (TRIzol reagent: Invitrogen, Carlsbad, CA, USA). Preliminary RNA samples were evaluated with the Agilent BioAnalyzer using the Nano RNA Kit. Only samples with RNA Integrity Number (RIN) > 7.0, and 5 total μg were used for library preparation, and two biological replicates were used for each age/breed group. The mRNA isolation was carried out by using the Dynabeads^®^ mRNA Direct^™^ kit (Thermo Fisher), and following recovery, polyA RNA was evaluated using the BioAnalyzer Nano mRNA assay, verify that samples contain less than 2.0% rRNA. For dUTP directional mRNA libraries preparation, 25-100ng mRNA was fragmented by chemical hydrolysis, converted to first strand cDNA with random hexamers, and second strand synthesized with dUTP according to the NEBNext Ultra Directional RNA library preparation Kit for Illumina (New England Bio Labs). The cDNA fragments were end-repaired, A-tailed, and ligated to the TruSeq y-tail single indexes from Illumina TruSeq DNA kit. Indexed libraries were cut with USER enzyme, and PCR amplified for 12 cycles. Finally, to achieve the highest quality data on Illumina sequencing platforms, optimum cluster deposition was made by quantitation of libraries using qPCR according to the Illumina Sequencing Library qPCR Quantification Guide (Kapa Biosciences). 156x156 bp paired-end sequence reads were generated using the Illumina NextSeq 500 platform High Output/300 cycle kits from Illumina.

### Bioinformatics analysis

Pre-processing analysis of RNA-seq data and sequence quality control (QC) was performed prior to read alignment to reference genome. For all libraries, the adaptor sequences were removed using the cutadapt software with minimum overlap length was set to 10 and error rate was set to 0.05 [40]. After adaptor trimming, the low quality bases were trimmed from 3’- end followed by sequence quality control (FastQC). The post processing analysis of RNA-seq data was performed i) to map the sequencing read (read alignment), as well as ii) to discover the breed-specific putative SNPs.

1. **Alignment of RNA-seq sequencing reads to the reference genome.** The pre-processed short paired-end reads were aligned to the reference genome Ensembl75_UMD3-1.1 plus the Chromosome Y from Btau_4.6.1 assembly, by using BWA version 0.7.5-r404 [41]. The HT-Seq framework, version 0.5.3p9 (https://pypi.python.org/pypi/HTSeq/0.5.3p7), was used to count the aligned reads in genes using the STAR BWA tools [42].
2. **Breed-specific discoveries of novel putative SNPs in Polish HF, Polish Red, and Hereford cattle.** For the analysis of SNPs discoveries (variant calling), the SAMtools mPileUp package to call SNPs and *indels* [34] was utilized to detect the putative breed-specific SNPs in bovine pituitary gland transcriptome.
  Using Microsoft Office Excel, the following two stringent parameters of SNP filtering were performed.
  - Stringent parameters of SNP filtering with minimum 10 SNP reads per base and with an accuracy of ≥ 90%.
  - Stringent parameters of SNP filtering with minimum 10 SNP reads per base and with an accuracy = 100%.
  Similarly, using Microsoft Office Excel the breed-specific SNP database of bovine pituitary gland were further trimmed to a highly reliable breed-specific SNP-db according to SNP type, *de novo* SNP and annotated SNPs, respectively.
3. **Breed-specific SNPs discoveries and QTLs/CGs analysis.** The publicly available animal quantitative trait loci (QTL) database (Animal QTLdb: http://www.animalgenome.org/cgi-bin/QTLdb/index) representing trait associated QTLs as well as candidate gene and association data (GWAS) mapped to bovine genomes was utilized to find putative SNPs within the candidate genes for bovine body growth and development trait [43]. A total of 76 potential QTLs/CGs (S29 Table) for bovine body growth and developmental trait was selected to investigate the RNA-seq SNP-db. To identify the putative SNPs within the CGs loci, stringent SNP filtering was carried out on both RNA-seq and bovine QTL databases using Microsoft Office Excel.
4. **Phylogenetic analysis.** The phylogenetic package [27] to call SNPs and *indels* was utilized to detect the putative breed-specific SNPs in bovine pituitary gland transcriptome. Phylogenetic analysis was based on the combined transcriptome datasets of all three breeds using Maximum likelihood method. An unrooted phylogeny tree of 18 bull’s samples representing CP01-CP06 (Hereford), CP07-CP12 (Polish Red) and CP13-CP18 (Polish-HF) was constructed by snp-phylo using SAMtools unified SNPs with following parameter “-c 3 -m 0.4 -a 30 -P CP1-18.snphylo -l 0.2 -m 0.2 -M 0.2”.

#### KASP^™^ assays design and SNP validation

Eight breed-specific SNPs (Polish HF) were selected for KASP^™^ SNP assays validation experiment. The primer sequences are presented in Table 6. The genomic DNA of 44 young bulls from Polish HF, Polish Red, and Hereford were isolated from pituitary gland tissue using the MasterPure^™^ DNA purification kit with some modifications (Epicentre). All 44 DNA samples were then shipped to LGC genomics Teddington, Middlesex, TW11-0LY, UK (http://www.lgcgroup.com) to perform the KASP^™^ SNP genotyping assay including primer design. For each of selected breed-specific SNP marker, two allele specific forward primers and a common reverse primer were designed for use in fluorescence based competitive allele-specific PCR assays. Primers designed were done by LGC genomics, UK, by blasting the region of +200bp and -200pb around the nucleotide variation using the PrimerPicker [44]. The KASP^™^ genotyping reaction and PCR thermocycling conditions were carried out according to manufacturer’s recommendations (LGC Genomics, UK). PCR reaction mixture was carried out in a 10 μl-volume consisting of 2.5 μl DNA (10 ng/μl), 2.5 μl 2X KASP^™^ Master mix v4.0, and 0.07 μl 72X KASP^™^ Primer mix. PCR amplification and fluorescent end-point genotyping was carried out in a LightCycler thermocycler. Finally, data was analysed using Kluster-caller software (LGC Genomics. UK) to identify SNP genotypes.

**Table 6:** SNP-ID and primer sequences of selected breed-specific SNPs of Polish HF cattle.

SNP-ID	SNP assay primer sequences
**BTA25_33248237**	TCAACTTATGGAAAT[G/C]AGTCAACAGAAGAG
**BTA16_81545856**	GAGGGCTGGGGGACC[T/C]GGTCGGGAGGCAGC
**BTA21_68782355**	GTGTCAAGATTACCT[T/C]CGTAGTTCTGGACC
**BTA23_9540557**	GCACATCTCAAGCAT[T/C]GAGATTTATGATCC
**BTA10_102942684**	GATCAGAGACTGCCC[A/G]AGATCGTCAAACAA
**BTA25_37378666**	CTCAAATCTTGAAAG[A/G]AGTGTAAGACATAA
**BTA4_77555734**	TCAGCCTTCTACTCC[C/T]TCAGGCAGCCAGGG
**BTA19_27086284**	TCCTTTTATGTACGG[G/A]TTAAAAAAGGAAAG

#### Statistical analysis

Using the Genepop software (http://genepop.curtin.edu.au/), the following statistical tests were carried out to validate the breed-specific SNPs.

1. Estimates of direct relationship between breed and detected SNP polymorphisms was performed by the use PROC MIXED SAS 9.2 package, with the age as a random effect.
2. A Fisher test was performed across all populations, and for each locus pair to compute an unbiased estimate of the P-value.
3. Genic and genotypic differentiation was tested for each locus for all populations, and for each population pair across all loci.
4. Significance of differences in allele frequencies between samples were determined by the Exact Fisher’s method and the Markov Chain procedure [28], and the Fisher combined test was computed as a global test over loci to determine the overall significance.
5. Deviation from Hardy-Weinberg equilibrium for each population was calculated by a probability exact test using a Markov Chain method [29] as implemented in Genepop.

## Supporting Information

S1 Table RNA-seq SNP-db of bovine pituitary gland tissue of young bull-1 of Hereford cattle aged 6 months. (XLSX)

S2 Table RNA-seq SNP-db of bovine pituitary gland tissue of young bull-2 of Hereford cattle aged 6 months. (XLSX)

S3 Table RNA-seq SNP-db of bovine pituitary gland tissue of young bull-3 of Hereford cattle aged 9 months. (XLSX)

S4 Table RNA-seq SNP-db of bovine pituitary gland tissue of young bull-4 of Hereford cattle aged 9 months. (XLSX)

S5 Table RNA-seq SNP-db of bovine pituitary gland tissue of young bull-5 of Hereford cattle aged 12 months. (XLSX)

S6 Table RNA-seq SNP-db of bovine pituitary gland tissue of young bull-6 of Hereford cattle aged 12 months. (XLSX)

S7 Table RNA-seq SNP-db of bovine pituitary gland tissue of young bull-7 of Polish Red cattle aged 6 months. (XLSX)

S8 Table RNA-seq SNP-db of bovine pituitary gland tissue of young bull-8 of Polish Red cattle aged 6 months. (XLSX)

S9 Table RNA-seq SNP-db of bovine pituitary gland tissue of young bull-9 of Polish Red cattle aged 9 months. (XLSX)

S10 Table RNA-seq SNP-db of bovine pituitary gland tissue of young bull-10 of Polish Red cattle aged 9 months. (XLSX)

S11 Table RNA-seq SNP-db of bovine pituitary gland tissue of young bull-11 of Polish Red cattle aged 12 months. (XLSX)

S12 Table RNA-seq SNP-db of bovine pituitary gland tissue of young bull-12 of Polish Red cattle aged 12 months. (XLSX)

S13 Table RNA-seq SNP-db of bovine pituitary gland tissue of young bull-13 of Polish HF cattle aged 6 months. (XLSX)

S14 Table RNA-seq SNP-db of bovine pituitary gland tissue of young bull-14 of Polish HF cattle aged 6 months. (XLSX)

S15 Table RNA-seq SNP-db of bovine pituitary gland tissue of young bull-15 of Polish HF cattle aged 9 months. (XLSX)

S16 Table RNA-seq SNP-db of bovine pituitary gland tissue of young bull-16 of Polish HF cattle aged 9 months. (XLSX)

S17 Table RNA-seq SNP-db of bovine pituitary gland tissue of young bull-17 of Polish HF cattle aged 12 months. (XLSX)

S18 Table RNA-seq SNP-db of bovine pituitary gland tissue of young bull-18 of Polish HF cattle aged 12 months. (XLSX)

S19 Table Construction of filtered/trimmed breed-specific SNP-db of bovine pituitary gland transcriptome. (XLSX)

S20 Table Analysis of breed-specific SNP-db using the SNP filtering criteria of a minimum 10 SNP reads per base with an accuracy ≥ 90%. (XLSX)

S21 Table Analysis of breed-specific SNP-db using the SNP filtering criteria of a minimum 10 SNP reads per base with an accuracy = 100%. (XLSX)

S22 Table Screening of SNP type and occurrence in the experimental breed-specific SNP-db. (XLSX)

S23 Table Identification of unique SNPs using 90% and 100% SNP filtering in Hereford cattle. (XLSX)

S24 Table Identification of unique SNPs using 90% and 100% SNP filtering in Polish Red cattle. (XLSX)

S25 Table Identification of unique SNPs using 90% and 100% SNP filtering in Polish HF cattle. (XLSX)

S26 Table Identification de novo SNPs in Hereford, Polish Red and Polish HF cattle using 90% and 100% SNP filtering criteria. (XLSX)

S27 Table Distribution of Identified de novo SNPs counts in the investigated young bulls on the basis of S26 Table. (XLSX)

S28 Table Annotation of SNPs in Hereford, Polish Red and Polish HF cattle. (XLSX)

S29 Table Lists of 76 CGs representing full names, UMD3.1 genome locations, chromosomal locations and web links at bovine QTL-DB. (XLSX)

S30 Table Identification of putative SNPs hits of RNA-seq data on 76 potential candidate genes from bovine QTL-db in Polish Red cattle. (XLSM)

S31 Table Identification of putative SNPs hits of RNA-seq data on 76 potential candidate genes from bovine QTL-db in Polish HF cattle. (XLSX)

S32 Table Identification of putative SNPs hits of RNA-seq data on 76 potential candidate genes from bovine QTL-db in Hereford cattle. (XLSX)

S33 Table Summary of identified putative SNPs hits of RNA-seq data on 76 potential candidate genes from bovine QTL-db in Polish Red cattle. (XLSM)

S34 Table Summary of identified putative SNPs hits of RNA-seq data on 76 potential candidate genes from bovine QTL-db in Polish HF cattle. (XLSM)

S35 Table Summary of identified putative SNPs hits of RNA-seq data on 76 potential candidate genes from bovine QTL-db in Hereford cattle. (XLSX)

S36 Table SNP-ID, genome locations, SNP mutations, SNP genes descriptions of selected SNPs specific to Polish HF breed in RNA-seq experiment. (XLSX)

S37 Table Fixed effect of breeds and developmental ages on validated SNPs markers Using REML mixed model procedure. (XLSX)

S38 Table Distribution of SNP genotypes in the investigated young bulls. (XLSX)

S39 Table Distribution and frequencies of SNP alleles in the investigated young bulls. (XLSX)

S40 Table Breed specific distribution of SNP genotypes in the investigated young bulls. (XLSX)

S41 Table Genetic differentiation of SNP alleles frequencies among investigated cattle breeds using the Fisher's exact Probability test. (XLSX)

S42 Table Genetic differentiation of SNP genotypes frequencies among investigated cattle breeds using the Fisher's exact Probability test. (XLSX)

S43 Table Hardy-Weinberg test for genetic differentiation of investigated SNP loci using the Markov chain method. (XLSX)

S44 Table Hardy-Weinberg test for genetic differentiation of investigated cattle breeds using the Markov chain method. (XLSX)

## Abbreviations

SNP: Single nucleotide polymorphism
RNA-seq: RNA sequencing
HF: Holstein-Friesian
KASP^™^: kompetitive allele-specific PCR
BWA: Burrows-Wheeler Aligner
BCM-HGSC: Baylor College of Medicine Human Genome Sequencing Centre
MAS: marker assisted selection
GAS: gene assisted selection
GS: genomics selection
QTL: Quantitative trait locus
BTA: Bos Taurus autosome
indel: insertions and deletions
SNP-db: SNP database
HWE: Hardy Weinberg equilibrium
NGS: next generation sequencing
RIN: RNA Integrity Number
QC: quality control
TMEM9: transmembrane protein 9
DDX56: DEAD (Asp-Glu-Ala-Asp) box polypeptide 56
RNF167: ring finger protein 167
CGs: candidate genes
GHSR: growth hormone secretagogue receptor
IGF2BP2: insulin-like growth factor 2 binding protein 2
GAD1: Glutamate decarboxylase 1
IGFBP2: insulin-like growth factor binding protein 2
IGFBP5: insulin-like growth factor binding protein 5
MSTN: myostatin
LEPR: leptin receptor
APOA2: apolipoprotein A2
SLC44A5: solute carrier family 44, member 5
NTNG1: netrin G1
GHRHR: growth hormone releasing hormone receptor
IGF2BP3: insulin-like growth factor 2 binding protein 3
IGFBP1: insulin-like growth factor binding protein 1
IgFBp3: insulin-like growth factor binding protein 3
LEP: leptin
NPY: neuropeptide Y
NSIG1: insulin Induced gene 1
DPP6: dipeptidyl aminopeptidase-like protein 6
HGF: hepatocyte growth factor
IGFBP6: insulin-like growth factor binding protein 6
IGF-I: insulin-like growth factor 1
WNT10B: wingless-type MMTV integration site family member 10B
MYF6: myogenic factor 6
MYF5: myogenic factor 5
IGFBP7: insulin-like growth factor binding protein 7
CCSER1: coiled-coil serine-rich protein 1
PKD2: polycystic kidney disease 2
NCAPG: non-SMC condensin I complex, subunit G
KCNIP4: kv channel interacting protein 4
CAST: calpastatin
NPM1: nucleophosmin
PROP1: PROP paired-like homeobox 1
IGFBPL1: insulin-like growth factor binding protein-like 1
ATP6V1B2: ATPase, H+ transporting, lysosomal 9kDa, V1 subunit B2
IGF2R: insulin-like growth factor 2 receptor
RFX6: regulatory factor X, 6
MAP3K5: mitogen-activated protein kinase 5
GTF3C5: general transcription factor IIIC, polypeptide 5
RALGDS: ras association
SDC1: syndecan 1
DNMT3B: DNA (Cytosine-5-)-methyltransferase 3 beta
GHRH: growth hormone releasing hormone
FOXA2: forkhead box A2
HNF4A: hepatocyte nuclear factor 4, alpha
PLAG1: pleiomorphic adenoma gene 1
NUCB2: nucleobindin 2
BTG4: B-cell translocation gene 4
MGC134087: Bos taurus UPF0686 protein C11orf1 homolog-like (LOC538766)
ATM: ATM Serine/Threonine Kinase
UCP3: uncoupling protein 3
IGFN1: immunoglobulin-like and fibronectin type III domain containing 1
CAPN2: calpain 2
PRDM16: PR domain containing 16
ZBED6: zinc finger, BED-type containing 6
LHX4: LIM homeobox 4
IGFLR1: IGF-like family member 1-like receptor
IGFL1: IGF-like family member 1-like
IGFBP4: insulin-like growth factor binding protein 4
IGF2BP1: insulin-like growth factor 2 binding protein 1
FASN: fatty acid synthase
SREBF1: sterol regulatory element binding transcription factor 1
CARTPT: CART prepropeptide
GHR: growth hormone receptor
PRLR: prolactin receptor
IGF1R: insulin-like growth factor 1 receptor
GHRL: ghrelin/obestatin prepropeptide
MST1R: macrophage stimulating 1 receptor
DLK2: delta-like 2 homolog
MC4R: melanocortin 4 receptor
NPC1: niemann-pick disease, type C1
XYLT1: xylosyltransferase I
SH2B2: SH2B adaptor protein 2
SIRT1: sirtuin 1
IGF2: insulin-like growth factor 2
CAPN1: calpain 1
VEGFB: vascular endothelial growth factor B
